# Mayer-Rokitansky-Kuster-Hauser Syndrome: Embryology, Genetics and Clinical and Surgical Treatment

**DOI:** 10.1155/2013/628717

**Published:** 2013-02-04

**Authors:** Alfonsa Pizzo, Antonio Simone Laganà, Emanuele Sturlese, Giovanni Retto, Annalisa Retto, Rosanna De Dominici, Domenico Puzzolo

**Affiliations:** ^1^Department of Gynaecological and Obstetrical Sciences and Reproductive Medicine, University of Messina, Via C. Valeria 1, 98125 Messina, Italy; ^2^Section of Histology and Embryology, Department of Biomorphology and Biotechnology, University of Messina, 1-98122 Messina, Italy

## Abstract

Mayer-Rokitansky-Küster-Hauser (MRKH) syndrome is a pathological condition characterized by primary amenorrhea and infertility and by congenital aplasia of the uterus and of the upper vagina. The development of secondary sexual characters is normal as well as that the karyotype (46,XX). Etiologically, this syndrome may be caused by the lack of development of the Müllerian ducts between the fifth and the sixth weeks of gestation. To explain this condition, it has been suggested that in patients with MRKH syndrome, there is a very strong hyperincretion of Müllerian-inhibiting factor (MIF), which would provoke the lack of development of the Müllerian ducts from primitive structures (as what normally occurs in male phenotype). These alterations are commonly associated with renal agenesis or ectopia. Specific mutations of several genes such as WT1, PAX2, HOXA7-HOXA13, PBX1, and WNT4 involved in the earliest stages of embryonic development could play a key role in the etiopathogenesis of this syndrome. Besides, it seems that the other two genes, TCF2 (HNF1B) and LHX1, are involved in the determinism of this pathology. Currently, the most widely nonsurgical used techniques include the “Frank's dilators method,” while the surgical ones most commonly used are those developed by McIndoe, Williams, Vecchietti, Davydov, and Baldwin.

## 1. Aim

This current paper of literature aims at investigating the most recent outcomes of studies related to Mayer-Rokitansky-Küster-Hauser syndrome, focusing mainly on the embryological and genetic profile, in order to lead future diagnostic and therapeutic studies.

## 2. Materials and Methods

### 2.1. Materials

A complete research of the literature has been carried out using keywords, such as Mayer-Rokitansky-Küster-Hauser. Database of PubMed and Cochrane have been used as sources, focusing the analysis on the studies that provided clinical evidence. The research has been extended to publications on American Society of Reproductive Medicine, Human Reproduction Journal, European Journal of Obstetrics and Gynaecology and Reproductive Biology, Gynecological Endocrinology, Orphanet Journal of Rare Disease, Fertility and Sterility, Journal of Medical Genetics, American Journal of Obstetrics and Gynaecology, The American College of Obstetricians and Gynaecologists, and other relevant ones.

### 2.2. Method

The research focused on a critical revision of data of studies about epidemiology, aetiopathogenesis, diagnosis and therapy of Mayer-Rokitansky-Küster-Hauser syndrome, with a particular reference to the study of genetic mutations and to the effects on embryological profile.

## 3. Definition

Mayer-Rokitansky-Küster-Hauser (MRKH) syndrome is characterized by a physiological development of the secondary sexual characters and by a normal female karyotype 46 XX, but with a congenital aplasia of the uterus and of two/third superior parts of upper vagina [1]. Schematically, we may distinguish between a simple syndrome, of first type (I), and complex syndrome, of second type (II). In the second type, other associated malformations are found out Müllerian duct aplasia Renal Dysplasia and Cervical Somite anomalies (MURCS) with renal unilateral agenesis, renal ectopia, or horseshoed kidney [2]; skeletal alterations with a particular reference to vertebral anomalies with Klippel-Feil syndrome, melted vertebras, and scoliosis [3]; anomalies of auditory system; only in some cases heart defects and syndactyly or polydactyly [2] are associated with it. During our experience, we have already reported 4 cases of MRKH syndrome, of which 3 cases are without skeletal and urinary tract abnormalities, and one case associated with absence of right kidney and ectopia of the left one [4]. Some studies [5, 6] assume two different syndromes that are an isolated form of congenital agenesis of uterus and vagina and a more generalised condition, in which the agenesis of uterus and vagina is an important and specific feature within a more complex syndrome. Besides, atypical groups and other acronyms to indicate other associations of malformations, as genital-renal-ear-syndrome (GRES) may be taken into account.

## 4. Epidemiology

Although Rokitansky syndrome or MRKH syndrome (Mayer-Rokitansky-Küster-Hauser) has been considered for a long time as a sporadic anomaly, it is a clinical condition widely proved with an incidence of one out of 4.500 women. The congenital absence of vagina and of the upper part of the uterus is the primary feature of this illness, but it is often associated with a renal unilateral agenesis or/and skeletal malformations [7]. The type I (or isolated type) characterized by a vaginal-uterus aplasia is statistically less frequent than complex type, and it does not have any racial predisposition. However, it is a congenital disorder, which could not be diagnosed up to adolescence or at the beginning of adulthood. Besides, a study carried out by Oppelt et al. in 2006, has noticed the typical form in 47% of cases, atypical form in 21% of cases, and the MURCS form in 32% of cases; from this, one can infer that associated malformations are considerably frequent, and they represent more than 1/3 of cases [8].

## 5. Embryology

The malformations in embryonic phase are anomalies typical of those of “embryogenesis period,” that is, during the first eight weeks of development. Conventionally, embryogenesis is divided into two phases: *blastogenesis* and *organogenesis*. During the blastogenesis, in the early 28 days of development, the domains of genic expression influence globally on the development of all parts of the embryo. The integrated and interdependent nature of the early development may contribute to explain defects that emerge in this phase, which are usually very serious and sometimes deadly. Embryogenesis phase, from 29th to 56th of development day, is defined as organogenesis, because during this period organs start to develop. The defects acquired during the organogenesis are usually more circumscribed than those of blastogenesis and generally affect a single organ without compromising the survival of a developing organism. Being stated that it seems to be essential to consider the embryological origin of various elements of the genitourinary system, in order to understand the pathogenesis of a genital malformation [9]. Around the 5th week of pregnancy, Müllerian ducts (or paramesonephric ducts) appear as developing structures and with different ways depending on the considered part. The caudal extremity of the ducts is destined to merge and to constitute superior 2/3rd parts of vagina and uterine cervix, the intermediate part melts and creates uterine body, while the upper portions maintain their own independence and, opening in the coelomic cavity (future peritoneal cavity), make fallopian tubes. In the same period, the renal system develops through the growth of urethral sketch, derived from Wolff's ducts (mesonephric ducts) within the mesenchyme of the metanephros. In similar times, the migration of the primordial germinal cells from the yolk sac leads to the formation of ovaries which arise from mesenchyme and from the epithelium of genital crest of the intermediate mesoderm, with organogenetic processes different from those of mesonephros; therefore, the anomalies of Müllerian ducts are not associated, generally, with anomalies of the ovary development [1]. MRKH syndrome, which represents 5–10% of genital anomalies, may be considered as a resultant of a failed development between the fifth and the sixth week of pregnancy and of a consequent fusion on the median line of Müllerian ducts, that in this condition are linked only to the caudal mesonephric ligament, destined to make the round ligament. Smooth dorsal bundle of muscles of the bladder and of the rudimental vagina is regularly shaped, because these arise, respectively, from Wolff's duct and from Gartner's duct. Therefore, agenesis or renal ectopia are commonly connected with these alterations [10–12].

**Figure 1:**
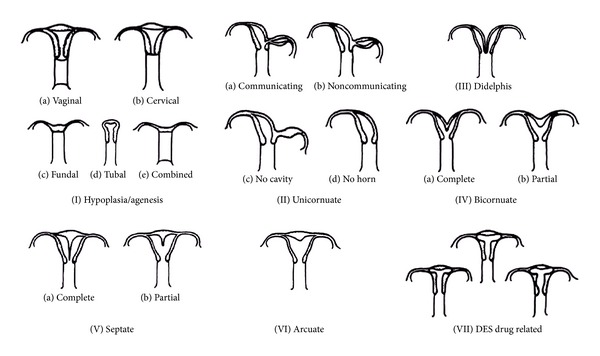
Classification of the anomalies of Müllerian duct developed by American Fertility Society (1988) and reproduced by Troiano and McCarthy [13].

Following the classification of uterine malformations adopted by the American Fertility Society (Figure 1), MRKH syndrome, as Troiano and McCarthy (2004) have pointed out previously, belongs to the first class [13]. This class assembles extensively the bilateral Müllerian agenesis or hypoplasias, and therefore, includes the vaginal agenesis, the agenesis of the neck and of the lower uterus, of tubes and the combined form, characterized by the agenesis of the body of uterus, which may be with two separated rudimental uterine sketches communicating with two normal developed tubes associated with vaginal agenesis, also known as Mayer-Rokitansky-Küster-Hauser. These malformations highly compromise the obstetric performance of a woman, and their treatment, when is possible, is not daily and encoded.

## 6. Aetiopathogenesis

Mayer-Rokitansky-Küster-Hauser (MRKH) syndrome has been considered for a long a time as an occasional anomaly, but the literature on familiar cases supports the assumption that a specific genetic substratum exists, and actually the syndrome seems to be transmitted as a dominant autosomal character with a penetrative incomplete capacity and variable expressivity [1]. Some studies investigate genetic mutations during the earliest phases of the embryonic development. There have been several assumptions about involved genes, such as Wilms tumor 1 (WT1), PAX2 (it is thought that the WT1 oncosuppressor may act as repressor of the transcription of PAX2), HOXA7–HOXA13 (highly important genetic clusters for the correct embryogenesis) [9], and pre-B-cell leukemia homeobox 1 (PBX1), although some research on direct implications of these genes has not given certain outcomes; the wingless-type MMTV integration site family, member 4 (WNT4) gene seems to be surely involved, since it intervenes on embryonic genital female development with a specific function [14–16].

Besides, other candidate genes have been reported, such as: TCF2 (also known as HNF1B, a gene that codifies a specific factor of transcription for the liver, belonging to the family of homeobox containing the double helix motive) and LHX1 (it produces a protein with the function of control factor for the development of nerve cells and lymphoid tissue). In a study [17] on 20 women suffering from MRKH syndrome, a screening has been carried out for the mutations of these genes, and it has been noticed that there were no alterations that were concerned with the pathology on current examination. Ledig et al. (2011), with the Array-CGH method, have identified three regions (1q21.1, 17q12, and 22q11.21), suggesting that LHX1 and HNF1B may be genes involved in the determinism of MRKH syndrome, having identified recurrent deletions and missense mutations [18]. Furthermore, in another study, some imbalances have been found which are concerned with chromosomal regions 1q21.1, 17q12, 22q11.21, and Xq21.31; LHX1 and KLHL4 are the candidate genes identified in this case (codifying gene for a member of family of Kelch proteins); the presence of the same alterations in a phenotypic normal mother of a patient has suggested the assumption of an incomplete penetrative capacity and/or variable expressivity [19]. Another study has shown that vaginal agenesis might be associated with a reduced activity of the galactose-1-phosphate uridyl transferase enzyme (GALT) [20]. The authors of this study assert that mutations of foetal or maternal GALT may cause a greater intrauterine exposition to galactose, which has been proved to be potentially harmful to the development of genitourinary system in mouse model. Ghirardini and Segre (1982) [21] have stressed and supported an assumption originally proposed by Schmid Tannwald and Hauser [22] according to it there would be a very strong hyperincretion of Müllerian-inhibiting factor (MIF) in patients with MRKH syndrome, which would cause a failed development of Müllerian ducts as primitive structures (as normally occurs in male phenotype) [23–25], and therefore, they have proposed that MRKH syndrome is considered as one of the slightest forms of pseudohermaphroditism. The hyperincretion of MIF could depend on a genes mutation previously described or on their altered expression, which provokes, therefore, a “*failure of maturation*” of structures deriving from Müllerian ducts. Besides, there is even the assumption that activator mutations in the gene for anti-Müllerian hormone (AMH) or in its receptor (AMHRII) may be considered as potential causes of the MRKH syndrome [26]. Moreover, there is an evidence that shows that partial duplication of pseudoautosomal Xpter region 1, containing homeobox gene for short height (SHOX), may be involved in the genesis of this current syndrome [27] (Table 1).

### 6.1. WNT4 Gene

WNT gene family is made up of structurally correlated genes, whose proteins are involved in different development processes, including cellular differentiation. Indeed, WTNT4 plays a key role both in the examination of female development and in the prevention of testicles formation. Jordan et al. (2001) have assumed that WNT4 is the first signal molecule which affects the cascade of events that culminates in sex determination, through local secretion of growth factors. Besides, they have proved that on laboratory animals a targeted deletion at this gene causes the occurrence of secondary male sexual characters in the offspring with XX karyotype; therefore, WNT4 may be considered as the responsible gene of the correct embryologic evolution of female sexual organs. Besides, overexposure of WNT4 leads to upregulation of DAX1 gene, which expresses in a female phenotype despite the occurrence of a XY karyotype. The conclusive assumption is that WNT4 may be the gene which determines sex and that DAX1 plays an important role, both in the checkup of female development and in the prevention of testicles formation [28]. Biason-Lauber et al. (2007) have found in R83C gene the loss of negative dominant function of WNT4 gene, concluding that Müllerian anomalies as lack of uterus and excess of androgens are pathognomonic signs of defects of WNT4 gene [29]. Philibert et al. (2008), analyzing young women with primary amenorrhea and lack of Müllerian ducts, have identified a L12P mutation within exon 1 of WNT4 gene, attesting that WNT4 may be involved in the regulation of the development of Müllerian ducts and in the biosynthesis of the ovarian androgens [30]. Kousta et al. (2010), on the basis of those assumptions previously mentioned, have identified four susceptible genes involved in the following phases of the ovarian development (WNT4, DAX1, FOXL2, and RSPO1) [31].

## 7. Diagnosis

### 7.1. Clinical

Generally, amenorrhea is the first symptom in women with a normal 46,XX phenotype and karyotype [32], physiology and normal ovarian anatomy, and lack of signs of androgens excess [33]. An external exam is useful to establish a normal puberty, secondary sexual characters, and the normality of external genitals. It is essential to focus on the anatomic study to diagnose MRKH syndrome and its typology: a complete aplasia of the uterus with two rudimental horns connected by a peritoneal fold and the occurrence of normal Fallopian tubes lead to a first type MRKH diagnosis [34]. The second type shows itself with asymmetrical and symmetrical uterine hypoplasia; besides, aplasia of one of the two horns or two rudimental horns different in size and tuberous malformations (hypoplasia or aplasia is typical of one or both tubes) is associated with this second type [35].

The second type syndrome may appear with the association with other anomalies of upper urinary trait, skeleton, auditory system, and seldom of heart defects, and it is named as Müllerian duct aplasia renal dysplasia and cervical somite anomalies (MURCS) [36]. Müllerian anomalies are often associated with PCO (polycystic ovary), so much that it might be that even PCO could have an embryonic defect as aetiopathogenesis; indeed, it has been noticed that patients with septum uterus or bicornuate uterus have a greater predominance of PCO [37]. Besides, some cases have been described, concerning a complete Müllerian agenesia with tubes and hypoplastic ligaments and lack of ovary, with 46 karyotype, ectopic, and battered kidneys. In our previous work related to 4 patients with MRKH syndrome, we reported that in the first case, the age at diagnosis was 15 years, 20 years in the second case, in the third case, the patient came to our attention at 23 years after completed the diagnostic workup in another hospital, and 12 years in the fourth case [4].

### 7.2. Differential Diagnosis

Differential diagnosis is considered with primary amenorrhea in which there are normal secondary sexual characters and with gonadal dysgenesis. It is essential, therefore, to verify primary amenorrhea, of normal sexual secondary characters of the congenital lack of uterus and vagina of vaginal atresia. Vaginal transversal septum and imperforate hymen are not indicative of Müllerian aplasia. Ultrasound is an important diagnostic tool to define carefully the pelvic anatomy [38, 39]. A differential diagnosis would be even applied to insensitivity to androgens syndrome (AIS), also known as testicular feminization syndrome (TFM), a disorder in which male hermaphroditism caused by gene mutations for the androgens receptor arises. A patient suffering from this syndrome is immune to androgens and has hypospadia, micropenis, and gynaecomastia.

### 7.3. Laboratory Analysis

It is necessary to carry out a genetic study for X chromatin and karyotype, the checkup of the functionality of endocrine system through the identification of plasmatic levels of stimulating follicle hormone (FSH), luteinizing hormone (LH), prolactin, estradiol, 17*β*-estradiol, and progesterone. It is fundamental, therefore, to make a checkup of plasmatic levels, which are usually normal, testosterone, delta-4-androstenedione, 17-hydroxyprogesterone, and dehydroepiandrosterone [40, 41].

### 7.4. Diagnostic Imaging

#### 7.4.1. Transvaginal Ultrasound

Transvaginal ultrasound is a simple diagnostic and noninvasive method, and following the usual procedure, it represents the first assessment survey when it is expected to have a suspicion of Müllerian anomalies; it is useful to reveal lack of uterus between the bladder and the rectum. Furthermore, this is a careful method for the diagnosis and the classification of uterine congenital anomalies [42] and effective to estimate the structure of rudimental uterine horns in Rokitansky syndrome [43]. Besides, recent developments of tridimensional ultrasound and magnetic resonance have improved the skill to diagnose carefully anomalies and complex malformations of female reproductive system [44]. Tridimensional ultrasound has been highly more effective in terms of sensitivity and specificity, compared with the performance of bidimensional ultrasound for the study of anomalies of female genital trait [45].

#### 7.4.2. Magnetic Resonance

Magnetic resonance (MR) is a noninvasive diagnostic method and represents more specific tools for the diagnosis than the ultrasound. Usually, this diagnostic method is applied when an ultrasound report is uncertain and incomplete, since failed identification with uterus clearness or Müllerian rudiments and of ovaries does not imply necessarily their lack. Therefore, MR has strengthen in such a way its role of assessment methodology of Müllerian duct anomalies (MDA). Indeed, MR reveals exactly some structures of female genital trait and, moreover, can give detailed images of intrauterine anatomy, sketching the external part of uterus in all levels of multiple scanning in a unique exam [46]. It has been proved carefully with 100% of sensitivity and specificity, recording a good deal with laparoscopy (*κ* = 0,55) and a great deal with identification of cavitation between magnetic resonance and intraoperative ultrasound [47]. A study of Mueller et al. (2007), carried out on 103 patients undergone to magnetic resonance for suspected Müllerian anomalies, has used for long uterine axis, T1-spin-echo weighing (SE) (TR/TE,500/10) axial scanning, and for short uterine axis, T2-SE weighing (5.000/80) sagittal scanning, concluding that an excellent agreement was made (*κ* = 0.8) between MR and clinical diagnosis of anomalies of Müllerian ducts, for uterus estimation lacked an agreement between MR and clinical diagnosis in 83 out of 103 patients, disagreement in 15 out of 103, a doubtful report in 5 out of 103 due to an uncertain diagnosis. Despite the excellent agreement between MR and clinical diagnosis, some cases of discrepancy have been noticed, and this depends mainly on the lack of a precise and integrated scheme of classification, and on a low familiarity with complex and rare entities, and finally due to an inadequate representation of some structures through MR [48]. Furthermore, it is necessary to focus on the frequency of radiological alterations of distal extremities of upper limbs associated with MRKH syndrome. A study carried out on 40 patients has underlined brachymesophalangy from the second to fifth finger (22/39 patients), little distal phalanx of first finger (22/39 patients), long proximal phalanx in the third and fourth finger (19/39 patients), and long metacarpus from the first to fourth finger (20/39 patients) [49].

#### 7.4.3. Laparoscopy

Laparoscopic survey is applied in case of doubtful diagnosis after the realization of noninvasive exams, already described and allows the precise definition of anatomical alterations typical of the syndrome [50]. This defines the exact morphology of uterus anomalies, tubes, and ovaries. This medical technique is used generally as a survey of the preparation for the surgical operation.

## 8. Surgical Therapy

### 8.1. Techniques

As previously stated, Müllerian anomalies besides concerning uterus and vagina may appear associated with a more complex frame, with involvement of gonadic tubes and urinary system. Concerning this, it is necessary to point out that these conditions need a careful preoperative analysis and very often an *ad personam *planning of a surgical operation. Repair methods of interested structures aim to rebuild a normal anatomy making a neovagina with band separation of rectum from urethrovesical space [51]. The most used surgical procedures are the following: McIndoe, Williams, Vecchietti, Davydov, Baldwin, and no-surgical technique of Frank. Original techniques have been refined and implemented, such as William's technique modified by Creatsas. In our experience, we have already reported 4 cases: in the first, the patient (15 years old) was sent to a specialist center for surgical reconstruction of the vagina (we do not know which technique was used), in the second case (20 years old), the patient has decided to postpone the operation only after trying to expand the retrohymenal cavity through sexual intercourse, in the third case (23 years old), the patient achieved a substantial improvement in the length and breadth of the vagina through sexual intercourse, and in the fourth case (12 years), the patient was first subjected to surgery with McIndoe technique and then, resulting a persistence of pelvic pain, underwent resection of the rudimentary uterine horns by laparoscopy. In the last case, after two surgical operation, there was still chronic pelvic pain (probably caused by the presence of residual endometrial tissue, not evidenced in the followup by TC and ultrasound), and so the patient underwent therapy with a continuous very low-dose of oral estroprogestins. Currently, the patient has a satisfying sexual life, with remission of the pain previously highlighted [4].

### 8.2. Frank's Method

This method is not surgical and aims at the creation of a neovagina by using dilators with large calibre and gradually greater length. This method is used by a patient, through self-management of insertion and maintenance of the “*intruder*” at level of vaginal fovea, at least, for 2 hours a day [53]. The best outcomes are achieved when there is already a retrohymenal pit not inferior than 2-3 centimetres [51]. Historically, Ingram reported a first trying to use the bicycle seat as rudimentary vaginal intruders [54], whereas D'Alberton and Santi report a success in 95% of the patients undergone a dilatation of retrohymenal pit using coitus [55]. Very interesting are the data presented by Motta and D'Alberton [56–58]. They analyzed 108 patients with MRKH syndrome from 1955 to 2003, of which 55 (mean age at diagnosis: 19; mean age at treatment: 22) underwent surgical procedure, whereas 53 (mean age at diagnosis: 17; mean age at treatment: 20) used the “functional method” to expand the retrohymenal pit. Referring to patient's opinion, they observed in the “functional method” group that 83% of patients were satisfied of the procedure. Moreover, they analyzed also the anatomical outcome of the neovagina in the “functional method” group and found that in 75% of the cases there was an optimal outcome (elastic walls; depth > 7 cm, width > 4 cm), in 13% an acceptable outcome (elastic walls, depth between 5–7 cm, 2–4 cm in width), and in 12% a poor outcome (rigid walls, depth < 5 cm, width < 2 cm). Summarizing, in this group using the nonsurgical technique, there were 83% of patient's satisfaction and 88% of anatomical successful outcome (optimal + acceptable). About the other group undergone to surgical treatment, there were 76% of patient's satisfaction and 68% of anatomical successful outcome. Regarding the “functional method,” there are two type of complications: urethral coitus [59] and vaginal prolapse [60–62].

### 8.3. McIndoe's Method

McIndoe's technique consists of three phases: the first provides the dissection of an appropriate space between the rectum and the bladder, the second provides the collocation of a flap of an autologous cutis, and the third consists of a continuous and extended dilatation by using vaginal intruders. First step is based on transversal cut of retrohymenal pit, with a following dissection of rectovesical space. Consequently, it is visible a median raphe, which once cut, allows to reach an under peritoneal tissue. At this point, a cut on fibres of pubic-rectal muscles is made in order to raise diameter to enter vagina. A flap made up of dermis and epidermis (usually taken out of gluteus) is applied on a support tissue; moreover, it fits in vagina for a necessary period to assure radication. Usually, this period lasts 7–10 days and is subsequently monitored to highlight possible necrotic areas [63].

### 8.4. William's Method

William's method arose as an alternative to McIndoe's technique and aims at creation of a peritoneal bridge in order to reconstruct a normal anatomy of vaginal channel. It begins with a shaped “U” cut, about 4 cm from external urethral orifice. Margins affected by this cut are only cutis, and subcutaneously muscle-fascial layer is saved. Subsequently, a second suture is applied to draw near subcutaneously fat and muscular plane to give a support to neovagina. At the end of this procedure, a 4-5 centimetres channel is extracted, which is generally raised in length and calibre by using intruders [63].

### 8.5. Vecchietti's Method

The main aim of Vecchietti's method is to make a neovagina from gradual stretching of vaginal cutis of a patient. This implies the insertion of an olive-shaped tool in vaginal dimple, which is linked to nylon traction threads that, after having crossed the pelvic peritoneum, go out through abdomen and are subsequently fixed to a device of progressive traction. The operation is carried out in laparoscopy. Nylon threads, once placed, are pulled softly before being linked to traction device that is placed on abdomen; threads are collocated on progressive traction, about 1 cm a day, for 7 days. On the seventh day, Vecchietti's device is removed, and the woman needs to use vaginal dilators for 30 minutes a day to keep vaginal length [63].

### 8.6. Davydov's Method

The main goal of Davydov's technique is to make a neovagina using patient's peritoneum as covering. The operation is made in laparoscopy. A peritoneal cut shaped “U” is made to create a flap and a laparoscopic rectovesical access; besides, a rectal probe is used to identify a correct dissection plane. According to this procedure, the peritoneum is stitched with vaginal edges, and the upper vagina is made with a suture of the superior portion of serous membrane of the large intestine. Afterwards, it is necessary to insert in the neoshaped vagina a soft vaginal mould. The mould *in situ* needs to be kept for 6 weeks, and then vaginal dilators have been kept for 30 minutes a day to maintain a suitable vaginal length [63].

### 8.7. Baldwin's Method

This method implies a great surgical operation, as well as risks typical of a surgical intestinal operation. This procedure, which is usually made in laparotomy, provides the sample of a segment from intestine of a length peer to about 10 cm–12 cm, with its vascular peduncle still unharmed; this segment is graft on pelvis making a neovagina with a closed proximal extremity. A perineal shaped “H” split is carried out, up to vestigial lamina between the bladder and the rectum; the intestinal transplant is vascularised from lower sigmoid artery. It does not need self-dilatation in postoperatory phase [64].

### 8.8. Creatsas's Method

Vaginoplasty with William's method modified by Creatsas is a simple and fast technique in which a perineal cutaneous flap is used to make a sac; at the beginning, the hymen is cut to avoid haemorrhages during the first sexual intercourse, afterwards a shaped “U” cut is made in the perineum, and subsequently, tissues are mobilized, and the margins of internal skin of the created flap are stitched together with reabsorbed stitches [65]. 

### 8.9. Evidence on Different Surgical Techniques

Jasonni et al. (2007) in a retroprospective study estimated the outcomes of self-dilatation technique and Williams and McIndoe's surgical procedures, just described, analyzing 104 cases of vaginal aplasia (data on examination were collected between 1977 and 2002, and patients' age was between 13 and 18). Data related to self-dilatation, in the followup at 6 months verified that in 41 patients the vaginal cavity measured 10–12 cm and measured 3–5  cm in 14 patients, while in 49 patients no any remarkable outcome was gained; these last 49 patients have been undergone successfully to surgical therapy: 14 with William's technique and 25 with McIndoe's technique. Applying this study, William's surgical technique should be represented as a choice when self-dilatation fails partially, whereas McIndoe's procedure and its variants should be used when self-dilation is totally failed [66]. De Souza et al. (1987), in a study on patients who have undergone to vaginoplasty surgery following McIndoe's technique, has noticed a statistically relevant relationship between the length of neovagina and the occurrence of dyspareunia, focusing, therefore, that a perfect surgical operation is necessary to avoid patient's unease during this long period [67]. A retrospective study carried out by Allessandrescu et al. (1996) [68] focuses on intraoperatory and postoperatory complications rate. On 201 cases of MRKH syndrome undergone to vaginoplasty, there were 2 perforations of the rectum (1%), 8 infections of the graft (4,0%), and 11 infections from the point of sample of the graft (5,5%). Giraldo et al. (1996) [69] believe that vaginoplasty is the surgical technique to adopt mostly, carried out getting bilaterally vulvoperineal fasciocutaneous flaps, also named “*Malaga flaps*.” These authors, concerning this technique, attest that it is a secure and reliable procedure, since vascularization under lateral edge of big vagina lips is well known; it is an easy method, because the rotation of vertical flap is easier than using farther flaps; the risk to damage neurovascular perineal superficial peduncle and Bartolini's gland is lower; the innervation is extended to external 2/3rd of artificial vagina; the outcome is acceptable from a functional and aesthetical profile, without the aid of dilators and obturators. Several authors, in many studies, instead, are more inclined to laparoscopic approach, which is more secure and advantageous to the dissection of vesical-rectal space and to an accurate suture of pelvic peritoneum than laparotomic surgical approach [70–72]. Besides, the case report of Panici et al. (2007) [73] was found very interesting, related to the transplant of human vaginal autologous mucosal membrane cultivated “*in vitro*,*”* grafted for vaginoplasty surgery as covering of neoshaped channel. It has been pointed out that tissue radication has been equal to 99% 7 days after the surgery, and followup in 1 month had mucosal of vaginal channel with a normal morphology. As regards to functional-anatomy reaction of neovagina, Belleannée et al. (1998) have analyzed 8 cases and noticed that superficial eosinophil cells, carefully observed by biopsy in vaginoplasty cases, showed a good reaction of neovaginal epithelium to hormonal variations and that the occurrence of Doderlein's bacillus proved that regional surrounding of neovagina was functionally similar to that of a normal vagina [74].

## 9. Discussion

Mayer-Rokitansky-Küster-Hauser appears more and more probably as a pathology with a complex and multifactorial aetiology; besides, genetic alterations, which affect embryological profile, contribute highly to its determination. Despite the major developments in reconstructive surgery, female patients seem to be weighed by a very disabling pathology under an anatomic, physiological and psychological profile. Moreover, new studies in genetic and embryological field have been carried out, in order to clarify etiology better and open up new possible therapeutic horizons.

## Figures and Tables

**Table 1:** Genes involved in MRKH syndrome, from “GeneCards: the human gene compendium” [52].

Gene	Chromosome	Cytogenetic band	Beginning	End	Size	Orientation
WT1	11	11p13	32,409,321 bp from *pter *	32,457,176 bp from *pter *	47,856 bases	Negative filament
WNT4	1	1p36.23–p35.1	22,443,798 bp from *pter *	22,470,462 bp from *pter *	26,665 bases	Negative filament
PAX2	10	10q24	102,505,468 bp from *pter *	102,589,698 bp from *pter *	84,231 bases	Positive filament
HOXA7	7	7p15.2	27,193,335 bp from *pter *	27,196,296 bp from *pter *	2,962 bases	Negative filament
HOXA13	7	7p15.2	27,235,022 bp from *pter *	27,239,725 bp from *pter *	4,704 bases	Negative filament
LHX1	17	17q12	35,294,499 bp from *pter *	35,301,912 bp from *pter *	7,414 bases	Positive filament
HNF1B	17	17cen–q21.3	36,046,434 bp from *pter *	36,105,237 bp from *pter *	58,804 bases	Negative filament
KLHL4	X	Xq21.3	86,772,715 bp from *pter *	86,925,050 bp from *pter *	152,336 bases	Positive filament
SHOX	X	Xp22.33; Yp11.3	585,079 bp from *pter *	620,146 bp from *pter *	35,068 bases	Positive filament
